# Explaining Job Search Behavior in Unemployed Youngsters Beyond Perceived Employability: The Role of Psychological Capital

**DOI:** 10.3389/fpsyg.2020.01698

**Published:** 2020-07-10

**Authors:** Maria Magdalena Fernández-Valera, Mariano Meseguer de Pedro, Nele De Cuyper, Mariano García-Izquierdo, Maria Isabel Soler Sanchez

**Affiliations:** ^1^Department of Psychiatry and Social Psychology, Faculty of Psychology, University of Murcia, Murcia, Spain; ^2^Research Unit of Occupational & Organisational Psychology and Professional Learning, KU Leuven, Leuven, Belgium

**Keywords:** psychological capital, job search, perceived employability, unemployed, youngsters

## Abstract

Job search seems to be a daunting task for youngsters in the Spanish labor market, unfortunately so given that it is the best predictor of getting a job even during economic crisis. Accordingly, it is vitally important to find resources that promote youngsters’ job search. The present study examines the effect of psychological capital on job search through perceived employability in a sample of Spanish unemployed youngsters. We analyzed data of 568 Spanish unemployed youngsters aged 16–29 years using structural equation modeling. Results showed that unemployed youngsters who possess high levels of psychological capital also perceive more control over job search which is directly connected with their job search intention. Surprisingly, analyses also showed that perceived employability is not an antecedent of job search. Instead, psychological capital seems to be a more beneficial resource for keeping unemployed youngsters engaged in job search in an adverse economic context.

## Introduction

Labor market entry is a critical phase in the life of youngsters, also in the formation of full citizens (Benedicto, 2013). This phase is characterized by turbulence, now more than ever: the International Labor Organization (ILO) states that “it is not easy to be young in the current job market,” alluding to the challenges young people face to obtain a job with decent conditions (International Labour Organization [ILO], 2013, p. 1). The situation in Spain is even more worrying: Spain leads the list of countries with highest youth unemployment rates, 34.4% in 2018 in the population between 15 and 24 (OECD, 2018), and informal work is quite common (Instituto de la Juventud [INJUVE], 2013). Job search has particular resonance in this group of still unemployed youngsters (Bronk et al., 2018). Job search behavior is the best predictor of obtaining a job in general (Kanfer et al., 2001; Saks, 2005; López-Kidwell et al., 2013). It is even more critical during economic downturns (Verick, 2012; Vuolo et al., 2013; Georgiou and Nikolaou, 2018).

Not feeling employable as a consequence of the economic context (De Battisti et al., 2016) may hamper job search (McQuaid and Lindsay, 2002; McArdle et al., 2007; Koen et al., 2013; Cifre et al., 2018): people act upon their perception of being employable (Forrier et al., 2015; Lo Presti and Pluviano, 2016) and conversely, they do not act when they perceive to be unemployable owing to outcome uncertainty (see e.g., Verbruggen and De Vos, 2019). Accordingly, it seems important to probe how perceived employability can be fed. Perceived employability is understood as the individual’s appraisal of his/her chance on a job in the labor market. Such appraisals are the result of contextual and individual features, including one’s psychological state (Chen and Lim, 2012).

We advance psychological capital as particularly relevant in this context for three reasons. First, it is defined as a mental positive state (Luthans et al., 2007), which in general has been linked to perceived employability (Berntson, 2008; Chen and Lim, 2012; Chiesa et al., 2018). Second, psychological capital, unlike other concepts, is considered a “state-like” resource. This implies that it can be developed through interventions (Luthans et al., 2007). Third, it has been advanced as a useful resource to help the unemployed to be engaged in job seeking (Chen and Lim, 2012; Afua et al., 2016; Lim et al., 2016; Georgiou and Nikolaou, 2018). Accordingly, we expect that unemployed youngsters with high levels of psychological capital are more likely to perceive themselves as employable and ultimately to engage in job search.

All in all, the main objective of this study is to analyze the relationship between psychological capital and job search through perceived employability among young unemployed people in Spain. We do so using the Theory of Planned Job Search Behavior and Vallerand’s Hierarchical Model of Motivation. A particular strength of this study is that we provide an innovative approach to deepen the analyses of how a positive mindset could help unemployed youngsters to be engaged in job search even when the economic context is clearly adverse.

### Contextual Features

The young population is most affected by the difficulties in the Spanish labor market. The problem of youth unemployment in Spain is clearly structural because: (1) The outdated education system does not offer effective responses to employers’ requirements; (2) There is a strong polarization between highly educated and less educated young people, which means that there is a relatively large group of people without secondary education; (3) During the economic crisis thousands of jobs in the construction sector or related sectors disappeared. Most jobs were occupied by lower educated youngsters; (4) The Spanish labor regulation has induced labor market duality. This has led young people to occupy precarious employment (Bacaria et al., 2015). The result is that youth unemployment rate is very sensitive to the economic cycle. That is, youth unemployment stays relatively high in periods of economic expansion but rises quickly during recession (Hernández et al., 2012; Gómez and Arévalo, 2013).

The implication is that young people’s access to the Spanish labor market is an arduous task. In this study, we seek ways to improve the unemployed youngsters’ job search process. Although previous research has advanced job search models, the role played by psychological capital and perceived employability in this population group and in economically adverse contexts is unexplored.

### Theoretical Framework and Hypotheses

**FIGURE 1:**
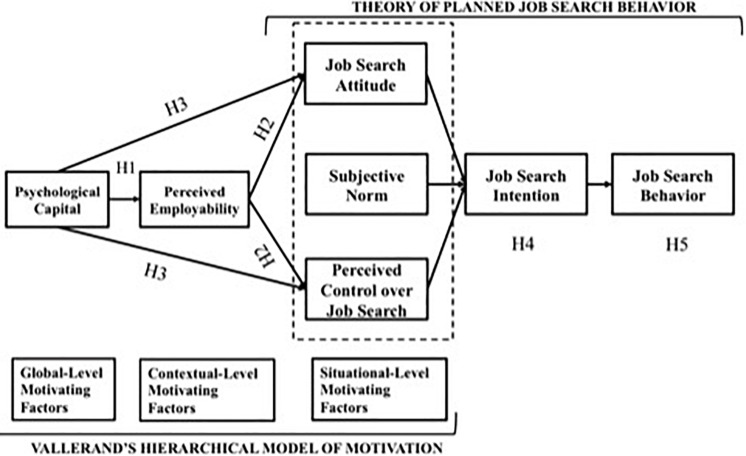
Conceptual model.

Grounded in Ajzen’s (2012) Theory of Planned Behavior, Van Hooft (2016) introduces the Theory of Planned Job Search Behavior as the result of integrating both motivational and self-regulatory perspectives on job search. This theoretical framework integrates the main mechanisms that are important in the job search process. On the one hand, as job search is a difficult and complex task, having and maintaining motivation is essential for securing continuous job search activities. On the other hand, as job search is not only difficult but also unpleasant, job seekers need self-regulation for ensuring task persistence and performance. The job search literature (Wanberg et al., 1999; Kanfer et al., 2001; Manroop and Richardson, 2015) has demonstrated the added value of including contextual-level and global-level motivators in predicting job search intention and behavior. In response, Van Hooft (2016) proposes Vallerand’s Hierarchical Model of Motivation as a useful frame to justify the extension of the Theory of Planned Job Search Behavior for analyzing which resources can help people to be engaged in job search. Based on both approaches, we have built an integrated conceptual model (Figure 1). In what follows, we describe the components of the model and how they are related.

Job search intention constitutes a central aspect in the Theory of Planned Job Search Behavior. Following Van Hooft (2016, p. 6), this component reflects the strength of an individual’s motivation to engage in job search. Job search behaviors are performed according to the intentions previously formed (Van Hooft and Noordzij, 2009; Van Hooft, 2016).

In order to understand how job search intentions and ultimately job search behaviors are shaped, Van Hooft (2016) proposes Vallerand’s motivational resources classification. This classification distinguishes between three hierarchical levels which vary in generality, stability and proximity to behavior, as follows: a) global-level motivating factors are generalized constructs that are stable over situations and are applicable to all life domains; b) contextual-level motivating factors concern specific life domains (e.g., employment); and, c) situational-level motivating factors refer to specific behaviors (e.g., job search). Vallerand recognizes a top-down direction from higher to lower levels. More specifically, motivating factors at proximal levels (e.g., from global level-motivating factors to contextual-level motivating factors) are more strongly related than motivational factors at a distal level (e.g., from global-level motivating factors to situational-level motivating factors) (Vallerand, 1997).

We propose psychological capital as a *global-level motivating factor* because it is not tied to a specific domain. It <psychological capital> is understood as a second-order construct composed of four components (optimism, resilience, hope and self-efficacy) and is defined as a state of positive psychological development characterized by having the confidence to face challenges and difficult tasks (self-efficacy); make positive attributions about present and future triumphs (optimism); visualize goals and persevere in pursuing goals, as well as redirect the objectives when necessary to achieve success (hope); and recover and even emerge stronger from adversity (resilience) (Luthans et al., 2007). In concert, those components relate to a tendency to hold cognitions and positive appraisals of one’s own ability (Luthans et al., 2007; Harms et al., 2018). Several studies have demonstrated that psychological capital has particular resonance in job search (Chen and Lim, 2012; Afua et al., 2016; Lim et al., 2016; Georgiou and Nikolaou, 2018). For example, Chen and Lim (2012) showed a positive association between psychological capital, perceived employability, and active job search. Lim et al. (2016) proposed psychological capital as a useful resource for reducing job search fatigue. Finally, Georgiou and Nikolaou (2018) argued that psychological capital helped individuals to persist in job seeking, which is linked to more job interviews, job offers, and reemployment. Psychological capital helps individuals to appraise job search in a more positive way, so that they find it easier to cope with stress, difficulties, and obstacles during their job search (Caska, 1998; Wanberg et al., 2005; Van Hooft, 2016).

We propose perceived employability, understood as the perceived ability to obtain sustainable employment appropriate to one’s qualification level (Rothwell et al., 2008), as a contextual-level motivating factor. It is tied to the employment domain with specific relevance to job search (Koen et al., 2013; Vanhercke et al., 2015; Lo Presti and Pluviano, 2016; Yizhong et al., 2017). For example, Koen et al. (2013) showed that employability improves not only the job search process but also long-term unemployed reemployment opportunities. Both Vanhercke et al. (2015) and Lo Presti and Pluviano (2016) suggested an association between perceived employability and well-being, and those job seekers with a better health status are more likely to carry out a successful job search (Crossley and Highhouse, 2005) and find a quality job (McKee-Ryan et al., 2005; Paul and Moser, 2009; Lim et al., 2016). Finally, Yizhong et al. (2017) argued that higher levels of perceived employability lead to higher levels of job search self-efficacy and job search intention in university graduates.

We advance job search attitude, subjective norm and perceived behavioral control over job search as *situational-level motivating factors.* Job search attitude is defined as the individual’s (positive or negative) evaluation of the job search process and job attainment (Van Hooft et al., 2004). According to the Theory of Planned Job Search Behavior, those job seekers with more positive attitudes toward job search will invest more time in looking for a job as they anticipate better results (Van Hooft, 2016).

Subjective norm is understood as the social pressure that an individual perceives when looking for a job. The Theory of Planned Job Search Behavior suggests that those unemployed who perceive a greater social pressure to look for a job from their immediate surroundings will dedicate more time to their job search process.

Perceived control over job search refers to people’s belief they can perform effectively when looking for a job. As a result, the more positive features people have of getting a job, and the stronger they value these features, the more likely they are to perceive job search as useful (Van Hooft, 2016). According to the Theory of Planned Job Search Behavior, these three variables are the most proximal antecedents of job search intention. Job search intention then is seen as the antecedent of job search behavior (Van Hooft, 2016).

Following Vallerand’s model, global-level motivating factors are expected to relate to contextual-level motivating factors and situational-level factors. This leads to the following set of hypotheses. First, we expect that psychological capital relates to perceived employability. This aligns with previous research that underlines the importance of personal resources and dispositions in fostering perceived employability (Berntson, 2008; Chiesa et al., 2018). Earlier studies support psychological capital as an antecedent of perceived employability (Chen and Lim, 2012; Afua et al., 2016; Chiesa et al., 2018). Accordingly, we hypothesize that:

Hypothesis 1. Psychological capital will relate positively to perceived employability.

Second, we hypothesize that perceived employability, as a contextual-level motivating factor, relates to job search attitude and perceived control over job search (situational-level motivating factors). Previous studies have suggested that perceived employability can help to understand how people appraise job search (Blau et al., 2013; Vanhercke et al., 2015), and this appraisal is captured in their attitude vis-à-vis job search. Perceived employability has been shown as a predictor of self-efficacy in several studies (Berntson, 2008; De Cuyper and De Witte, 2010; Vanhercke et al., 2015; De Battisti et al., 2016), and self-efficacy is often used as an operationalization of control over job search (Van Hooft et al., 2004). Note that we have not hypothesized any relation between perceived employability and subjective norm because there is little empirical basis for doing so (e.g., see Yizhong et al., 2017). The idea that perceived employability and subjective norm are unrelated is plausible: perceived employability does not need to be related to how significant others youngsters (e.g., parents, partner, friends, …) think about their job search.

Hypothesis 2. Perceived employability will positively predict job search attitude and perceived control over job search.

Third, we expect that psychological capital relates to job search attitude and job search control through perceived employability. This aligns with the idea that global-level motivating factors are distal to situational-level factors, and the relationship between them is indirect through contextual-level factors. This results in the following hypothesis:

Hypothesis 3. Perceived employability will mediate the relationship between psychological capital and job search attitude and perceived control over job search.

Finally, the Theory of Planned Job Search Behavior argues that job search attitude, subjective norm and perceived control over job search are directly related to job search intention and mediators of the relationship between other variables and job search intention (Van Hooft, 2016). In line with this argument, on the one hand, job search attitude and perceived control over job search will mediate the relationship between perceived employability and job search intention. On the other hand, as we have not hypothesized any relationship between perceived employability and subjective norm, we expect a direct association between subjective norm and job search intention. According with Van Hooft (2016) the more social pressure from important other job seekers perceive, more time they are likely to spend on their job search, as this could positively affect their relationship with significant others. In addition, job search intention will relate to job search behaviors, since changes in intentions will be followed by changes in behavior (Ajzen, 2012). Accordingly, we hypothesize that:

Hypothesis 4. Perceived employability will be related to job search intention through the mediating role of job search attitude and perceived control over the job search. Subjective norm will be directly related to job search intention.
Hypothesis 5. Job search intention will be positively associated with job search behavior.

## Materials and Methods

### Data Collection Procedure and Sample

Data were collected in Spain in collaboration with the public employment service (Employment and Training Service of the Region of Murcia, SEFCARM) between January and June 2016. Labor counselors invited unemployed youngsters job seekers aged between 16 and 29 to enroll in our study during their counseling trajectory. This study was carried out in accordance with the recommendations of ethics committee of Universidad de Murcia. All subjects gave written informed consent in accordance with the Declaration of Helsinki.

The sample included 568 young unemployed people with an average age of 23.4 years (*SD* = 3.79, range = 16–29 years). The large majority of the respondents were Spanish (91.1%). About half of the respondents (52.4%; *N* = 300) were men. Most respondents were single (81.8%; *N* = 469). The others (17.5%; *N* = 100) were married or lived together. In terms of education, 33.5% (*N* = 192) had university studies, 27.2% (*N* = 156) completed secondary education, 19.9% (*N* = 114) completed primary education and 18.8% (*N* = 108) followed Basic Vocational Training. Most respondents were looking for a job after one or several previous jobs (46.2%; *N* = 265), followed by those who were studying and looking for work at the same time (37.9%; *N* = 217) and those who were looking for their first job (14.8%; *N* = 85). Lastly, the average time in unemployment was 8.47 months approximately (*SD* = 2.24).

### Measures

Data were obtained using instruments previously validated in Spanish and similar populations.

Job Search. It includes five subscales based on previous research carried out by different authors and validated in Spanish by Fernández-Valera (2018). Participants had to rate the items on a scale ranging from 1 (*nothing*) to 5 (*a lot*).

(A) Job search behavior. We used the scale proposed by Van Hooft and Noordzij (2009). This scale is composed of eight items. Participants were asked how much time they have devoted to each of the job search activities in the last 4 months. Job search activities included, for example, contacting job placement agencies or looking for a job on the Internet. An example item is: “How much time did you spend on the following job search activities in the last 4 months: Looking for jobs on the internet” (Item 8). The coefficient α for this scale was 0.80.

(B) Job search intention. We used the same eight items from the previous scale, yet adapted to reflect intention. An example item is: “How much time do you intend to spend talking to friends or family about possible job opportunities in the next 4 months” (Item 2). The coefficient α for this scale was 0.85.

(C) Job search attitude. Van Hooft et al. (2004) distinguish between instrumental and affective job search attitude. We used only instrumental job search attitudes as this seems most relevant in a context of crisis and following first analyses (Fernández-Valera, 2018). The three items for instrumental job search attitude are based on the work of Vinokur and Caplan (1987). They concern the degree to which job seekers consider job search sensible, prudent and useful taking into account the next 4 months. An example of an item would be: “It is wise for me to search for a (new) job in the next 4 months.” The coefficient α for this scale was 0.65.

(D) Subjective norm. Based on the work of Vinokur and Caplan (1987), subjective norm was evaluated using two items. The participants were asked to indicate how much they perceive their environment thinks they should look for a job in the next 4 months. For example: “My significant other thinks that I should search for a (new) job in the next 4 months.” The coefficient α for this scale was 0.90.

(E) Perceived control over job search. This refers to the perception of control over both internal and external resources that may influence job search. We included perceived control over internal resources as this seems most relevant in a context of economic crisis, which found support in preliminary analyses reported by Authors (Fernández-Valera, 2018). Perceived control over internal resources included eight items based on the previous work of Ellis and Taylor (1983) and Van Ryn and Vinokur (1992). An example of an item is: “I trust that I can offer a good impression in job interviews.” The coefficient α for this scale was 0.64.

Psychological Capital. The OREA questionnaire developed by Meseguer-de Pedro et al. (2017) was used to measure psychological capital. It consists of 12 items, three items for each dimension of psychological capital. The items of OREA questionnaire are rated on a four-point scale, that is, going from 1 (*strongly disagree*) to 4 (*strongly agree*). Sample items per dimension are: “In difficult times I usually expect the best” (Optimism); “I usually reach my goals even if there are obstacles” (Resilience); “I think my life is worth it” (Hope); “I am confident about I could effectively handle unexpected events” (Self-efficacy). The coefficient α for this scale was 0.82.

Perceived employability. Perceived employability was measured using three items from the Employment Outlook Scale of Career Exploration Survey (Stumpf et al., 1983), validated in Spanish by Ripoll et al. (1994). Responses were made using a five-point scale that varies from 1 (*strongly disagree*) to 5 (*strongly agree*). A sample item is: “It is possible for me to find a job in a company of my choice.” The coefficient α for this scale was 0.81.

Control variable. As there were diverse groups in our sample (e.g., those who were looking for a first job, those who had worked before and those who was looking for work and studying at the same time) who might have different perceptions about labor market access, we controlled for current situation (0 = First job; 1 = Previous job; 2 = Studying and looking for a job). We test hypotheses with and without control variables, with virtually similar results. We report results without control variables following the principle of parsimony (Vandekerckhove et al., 2015).

## Results

### Descriptive Analyses

We first inspected descriptive statistics using the Statistical Package for the Social Sciences Version 22.0. Table 1 shows the means, standard deviations, and correlations of variables included in this study.

**TABLE 1:** Means, Standard Deviations, and correlations between the study variables.

	*M*	*SD*	1	2	3	4	5	6	7
1. Job search behavior	3.04	0.83	(0.80)						
2. Job search intention	3.43	0.78	0.62**	(0.85)					
3. Instrumental job search attitude	3.67	0.65	0.13**	0.30**	(0.65)				
4. Subjective norm	4.10	1.01	0.30**	0.36**	0.26**	(0.90)			
5. Perceived control over job search	3.81	0.64	0.28**	0.37**	0.14**	0.17**	(0.64)		
6. Psychological capital	2.71	0.90	0.06	0.13**	0.09*	–0.01	0.45**	(0.82)	
7. Perceived employability	3.15	0.40	–0.03	–0.02	0.04	–0.01	0.17**	0.25**	(0.81)

Cronbach’s alphas appear in parentheses along the diagonal. n = 568. ** p < 0.01; * p < 0.05.

### Structural Equation Modeling

We then used Mplus 7 to test the hypotheses (Asparouhov and Muthén, 2012) applying a maximum likelihood estimation. Models were evaluated using the Comparative Fit Index (CFI), Tucker Lewis Index (TLI), Root Mean Square Error of Approximation (RMSEA), Standardized Root Mean Square Residual (SRMR), and Akaike Information Criterion (AIC) fit indices, with conventional standards.

We first performed a confirmatory factor analyses for all the variables simultaneously. The seven-factor measurement model in which psychological capital is a second order factor provided an adequate fit to the data [χ^2^/(df) = 2.32, *p <* 0.01, *CFI* = 0.91, *TLI* = 0.90, *RMSEA* = 0.05, *SRMR* = 0.07; AIC = 43714.02]. This model provided a better fit compared to a model in which psychological capital is a single first-order factor, χ^2^/(df) = 2.11, *p <* 0.01, *CFI* = 0.89, *TLI* = 0.87, *RMSEA* = 0.04, *SRMR* = 0.07; AIC = 491123.68.

**FIGURE 2:**
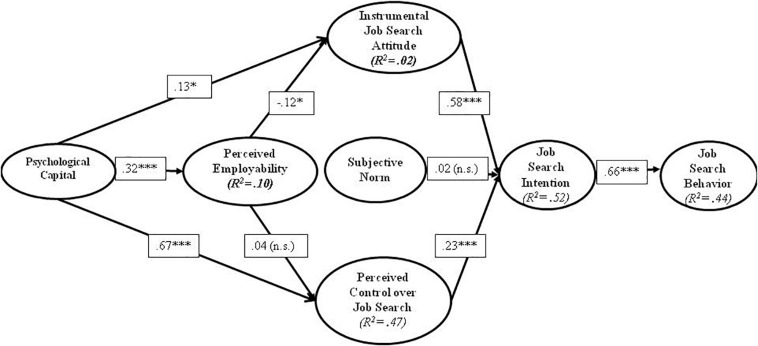
Structural model. Standardized results.

We then used structural equation modeling with latent variables to test our hypotheses: We modeled paths as shown in Figure 1, however goodness of fit indices of were not satisfactory [χ^2^/(df) = 3.34, *p <* 0.01, *CFI* = 0.84, *TLI* = 0.82, *RMSEA* = 0.07, *SRMR* = 0.06]. We inspected the modification indices and added the following correlations: following the Theory of Planned Job Search Behavior (Van Hooft, 2016) we added correlations between the antecedents of job search intention, namely, (1) job search attitude and subjective norm; (2) job search attitude and perceived control over job search; (3) subjective norm and perceived control over job search. Furthermore, we also added correlations between items of job search behavior and job search intention, as those items were largely similar (see Appendix Table A1): in particular, we connected (4) items 5 of job search behavior and 7 of job search intention; (5) items 1 of job search behavior and 8 of job search intention; (6) items 6 of job search behavior and 9 of job search intention; (7) items 7 of job search behavior and 10 of job search intention. The hypothesized model provided a reasonably good fit to the data [χ^2^/(df) = 1.70, *p <* 0.01, *CFI* = 0.95, *TLI* = 0.94, *RMSEA* = 0.04, *SRMR* = 0.04] (see Figure 2).

Hypothesis 1 stated that psychological capital is positively associated with perceived employability. This hypothesis found support (β = 0.32; *p <* 0.001).

Hypothesis 2 stated that perceived employability related positively to instrumental job search attitude and perceived control over job search skills. Perceived employability was weakly and negatively related to instrumental job search attitude (β = −0.12; *p <* 0.05) and not significantly related to perceived control over job search (β = 0.04; *p* > 0.05). Hence, Hypothesis 2 was not supported.

With respect to Hypotheses 3 and 4 concerning mediation, we applied the bias-corrected (BC) bootstrapping confidence interval (CI) analyses with 1000 bootstrap sample in Mplus (Preacher and Hayes, 2008). Using BC bootstrapping CI analyses, we construct 95 per cent confidence intervals of our tests.

Hypothesis 3 concerned perceived employability as a mediator in the relationship between psychological capital and both job search attitude and perceived control over job search. The indirect effect of psychological capital through perceived employability on job search attitude (β = −0.04, SE = 0.02, *p* = 0.076) and on perceived control over job search (β = 0.01, SE = 0.02, *p* = 0.519) was not significant. Hence, Hypothesis 3 was not supported (see Table 2). Hypothesis 4, on the one hand, argues perceived employability is related to job search intention through job search attitude and perceived control over job search, on the other hand, states a direct relationship between subjective norm and job search intention. There was no significant direct effect between perceived employability (β = −0.01, SE = 0.04, *p* = 0.722) and subjective norm (β = 0.02, SE = 0.07, *p* = 0.742) with job search intention. In addition, the indirect effect of perceived employability on job search intention through job search attitude (β = −0.07, SE = 0.04, *p* = 0.073) and perceived control over job search (β = 0.01, SE = 0.01, *p* = 0.542) was not significant. The results show that neither instrumental job search attitude neither perceived control over job search mediate the relationship between these variables (see Table 2). Lastly, and in line with Hypothesis 5, job search intention was a significant and positive antecedent of job search behavior (β = 0.66, SE = 0.04, *p* = 0.000).

**TABLE 2:** Mediational hypotheses.

Hypothesis	Direct effect	Total effect	Indirect effect	(I.C. 95%)	(I.C. 95%)	Sobel test	*p*
P.C. → P.E. → I.A.	0.13*	0.10 (n.s.)	−0.03 (n.s.)	−0.05	−0.01	−2.01	0.055
P.C. → P.E. → P.C.S	0.73***	0.71***	0.01 (n.s.)	−0.01	0.04	1.25	0.209
P.E.→ I.A. → INT.	0.02 (n.s.)	−0.02 (n.s.)	−0.07 (n.s.)	−0.06	0.01	−0.83	0.069
P.E. → P.C.S. → INT.	−0.08*	−0.02 (n.s.)	0.01 (n.s.)	0.03	0.11	3.76	0.000

Bootstrap analysis and Sobel test. P.C., Psychological Capital; P.E., Perceived Employability; I.A., Instrumental Job Search Attitude; P.C.S., Perceived Control over Job Search; INT., Job Search Intention. *p < 0.05; *** p < 0.001.

## Discussion

Job search seems to be a daunting task for youngsters in the Spanish labor market. Particularly in times of an adverse economic backdrop, job seekers face more obstacles, setbacks and rejection that can undermine their motivation (Van Hooft, 2016). Besides, today’s young people, also known as the Millennials generation, are searching for a job not only amongst a challenge economic time but also a greater life transition when trying to adjust to a new way of living and financial situation, whereas simultaneously manage changing personal relationships (Smith, 2018). Therefore, it is critical to identify those resources that promote job search in young people as a first step to overcoming difficulties. Using both Vallerand’s Hierarchical Model of Motivation and the Theory of Planned Job Search Behavior, we propose an integrative model to examine the effect of psychological capital on job search through perceived employability in a sample of Spanish unemployed youngsters.

Analyses revealed that psychological capital associated positively with perceived employability (Hypothesis 1); Perceived employability was negatively yet weakly associated with instrumental job search attitude and not associated with perceived control over job search (Hypothesis 2); Perceived employability did not mediate the relationship between psychological capital and instrumental job search attitude or perceived control over job search (Hypothesis 3); Instrumental job search attitude and perceived control over job search did not mediate the relationship between perceived employability and job search intention, and subjective norm did not have a significant effect on job search intention (Hypothesis 4); Job search intention was positively associated with job search behavior (Hypothesis 5).

This pattern of results lent partial support for the extension of the Theory of Planned Job Search Behavior with Vallerand’s Hierarchical Model of Motivation. Following the top-down direction proposed in Vallerand’s Model, psychological capital (global-level motivating factor) was positively associated with perceived employability (contextual-level motivating factor). This argument supports recent studies that have shown similar top-down effects between global and contextual-level motivating factors both in the academic (Núñez and León, 2015) and health domain (Miao et al., 2016). Also, this finding supports studies that showed psychological capital as an antecedent of perceived employability during job search (Chen and Lim, 2012; Afua et al., 2016; Ngoma and Dithan Ntale, 2016; Chiesa et al., 2018).

However, the relationship between perceived employability as a contextual-level variable and job search attitude and control over job search as situational-level variables was not in line with our hypotheses. First, perceived employability is weakly but negatively related to job search attitude. A possible explanation is that people try to invest the minimal necessary effort and time when looking for a job, as job search is an activity more likely to be driven by results than by pleasure (Van Hooft, 2016). This argument suggests that job seekers with high expectations about the ease of finding a job may perceive that a job offer is likely also with lower effort (Sun et al., 2013; Van Hooft, 2016).

Second, perceived employability was not related to perceived control over job search, while we expected a positive relation, also based on previous findings (Berntson, 2008; De Cuyper and De Witte, 2010; Vanhercke et al., 2015; De Battisti et al., 2016). As we explain below, it could be related to the strong role of psychological capital as antecedent of perceived employability and perceived control over job search.

Vallerand (1997) states that there are specific conditions that may lead motivation at the global level to have a direct impact on motivation at the situational level without involvement of contextual levels. One such condition is when there is no perceived relationship between task and contextual motivation. In an adverse economic context, such as the Spanish economy, it’s highly possible that an unemployed youngster does not perceive a relation between the context (less job offers) and the task (job search behavior). Indeed, they may think job search is useless when there are no jobs available. Vallerand (1997) furthermore advances the notion of “compensation effect”: losses of motivation in one life context (for example, employment) can be compensated by motivational gains in another life context (for example, life domain context). In that way, motivation losses in one life context are countered by gains in other life contexts in order to restore a general equilibrium in the self (Vallerand, 1997).

Interestingly, the findings suggest that perceived employability is less useful in this context. It could be speculated that global-level motivating factors, such as psychological capital, are more important for young people than contextual-level motivating factors, such as perceived employability. Young people lack work experience, as well as skills required to obtain jobs (Fallows and Steven, 2000; Smith, 2019) and aspects related to work (e.g., perceived employability), are not yet a central aspect in their lives. If so, their global mindset (e.g., psychological capital) acquires particular resonance. In line with this argument, Koen et al. (2013) argued that employability plays a very small role in the reintegration of long-term unemployed people. Instead, it is more important that job seekers remain energetic and committed to the job search process. Following this argument, it is possible that perceived employability does no matter to explain the success or failure of their job search for unemployed youngsters, and psychological capital as a global and positive cognitive appraisal is more beneficial for them.

We have not validated the mediational hypotheses which is not suprising given the results related to perceived employability. Our results are in line with those presented by Van Hooft et al. (2004), who did not find support for the idea that the effect of other variables (job satisfaction, organizational commitment, work valence, expectancy, and financial need) on behavior is completely mediated by the variables of the Theory of Planned Behavior. Also, we have no found a direct association between subjective norm and job search intention even when it seems to be a stronger predictor of intentions in job seeking context compared with the general one. Following Van Hooft (2016), a possible explanation could be that for unemployed job seekers, finding a job is important not only for themselves but also for their partners or children (subjective norm). As our sample is composed of youngsters, family care is probably not a big issue in their lives yet.

Lastly, job search intention is significantly and positively associated with job search behavior. This finding provides support for the Theory of Planned Job Search Behavior.

### Implications for Theory and Practice

The results of this study have implications for young job seekers and employment services. The International Labour Organization (ILO) (2015) highlights that persistently high youth unemployment rates have long-term consequences, for example skills erosion, increase of social exclusion and a higher level of poverty. Furthermore, the experience of unemployment at the beginning of professional life leaves scars, for example in terms of future salary (Bell and Blanchflower, 2011), job and personal satisfaction (Morsy and Jaumotte, 2012) and long-term unemployment (Arulampalam et al., 2001). We demonstrated that psychological capital may facilitate job search as a way to overcome unemployment.

Regarding implications for theory, the results show partial support for the extension of the Theory of Planned Job Search Behavior through Vallerand’s Hierarchical Model of Motivation. Perhaps this is because the specific population group (unemployed youngsters) and the context (Spanish economy) we have analyzed. Regarding practical implications, both public and private employment services could design intervention programs aimed at increasing unemployed’s psychological capital. Inspiration can be found in the study by Georgiou and Nikolaou (2018) who showed that psychological capital can be developed among greeks job seekers following Luthans et al.’s (2007) PCI (Psychological Capital Intervention) training model.

### Future Research

As regards the proposed model, to avoid the need of adding correlations between very similar wording items, further research is needed to probe its strength with different job search intention and job search behavior measures. For example, previous research (Song et al., 2006; Wanberg et al., 2010; Yizhong et al., 2017) have applied Vinokur and Caplan (1987) 2 item scale for job search intention and Wanberg et al. (1999) 12 item scale for job search behavior. Further investigation could extrapolate the proposed model to other populations and contexts. For example, in NEET (Not in Education, Employment or Training) population, unemployed people over 45 years or long-term unemployed people, also in favorable economic contexts. Besides, we have not hypothesized any relation between perceived employability and subjective norm, even when the social network is considered one of the components of some employability approaches (Lo Presti et al., 2019). Future studies may put more emphasis on subjective norms as previous research has reported an association between the fact of receiving helpful and supportive messages from their close environment and job search intensity in Millenials (Holmstrom et al., 2013; Smith, 2019). Finally, according to Smith (2019) a common millennial generation job search strategies are characterized by both the use of interpersonal networking and the use of online channels. As a result, further research would also benefit from focusing on variables related to social networks (for example, regarding our proposed model, social capital as a global motivating factor and job contact network as a situational motivating factor) and the analysis of the influence of technology on the traditional job search landscape.

### Limitations

Our study has some limitations. First, we used self-ratings. Though commonly used in job search research, this may induce common method variance, which could lead to inflated relationships (Spector, 2006).

Second, we measured job search behaviors in terms of intensity and frequency. While this is commonly done, job search behaviors are likely more complex and multidimensional. Future research may want to focus upon job search methods, job search quality and job seekers’ procrastination during job search (Van Hooft et al., 2012).

Third, our study was cross-sectional. This means that we cannot make causal conclusions. Yet, our hypotheses are in line with earlier work and grounded in theory.

Fourth, in the Structural Equation Model we have added correlations between items from the job search behavior and intention scales. This was done owing to very similar wording across the ítems, yet admittedly a cause for concern.

## Conclusion

Our overall conclusion is that psychological capital is a useful resource to facilitate job search among unemployed youngsters. Psychological capital helps young people to persist in job search even when the economic context is clearly hostile. Job seekers who posses high levels of psychological capital have a higher perception of control over their job search process which is directly link with the amount of time they expend looking for a job. In conclusion, our findings add to the growing evidence on the importance of personal resources in promoting the persistence in job search.

## Data Availability Statement

The datasets generated for this study are available on request to the corresponding author.

## Ethics Statement

The studies involving human participants were reviewed and approved by the Ethics committee of the Universidad de Murcia. The participants provided their written informed consent to participate in this study.

## Author Contributions

MF-V was responsible of designing the study, collecting the data, performing the data analysis, and writing the first draft of the whole manuscript. MM supported the data analysis, data collection, and the “Discussion” section. ND reviewed and adapted the structure of the “Introduction” and “Results” sections, contributed with greater theoretical strength to the arguments, and included some employability literature. MG-I and MS were responsible of the theoretical framework and revising repeatedly all the different version of the manuscript. All authors contributed to the article and approved the submitted version.

## Conflict of Interest

The authors declare that the research was conducted in the absence of any commercial or financial relationships that could be construed as a potential conflict of interest.

## Appendix 1

### Measures of Job Search Behavior and Job Search Intention

**TABLE A1:** Job search behavior and job search intention subscales.

**Job Search Behavior.** How much time did you spend on the following job search activities in the last 4 months:	

1. Looking for jobs on the internet?	1 2 3 4 5
2. Reading classified or help wanted advertisements?	1 2 3 4 5
3. Talking with friends or relatives about possible job leads?	1 2 3 4 5
4. Speaking with previous employers or business acquaintances about possible job leads?	1 2 3 4 5
5. Contacting employment agencies?	1 2 3 4 5
6. Making inquiries to prospective employers?	1 2 3 4 5
7. Sending out application letters or filling out job applications?	1 2 3 4 5
8. Preparing of and going on job interviews?	1 2 3 4 5

**Job search intention.** How much time do you intend to spend on the following job search activities in the next 4 months:	

1. Making inquiries or reading about getting a job?	1 2 3 4 5
2. Preparing or revising your resume?	1 2 3 4 5
3. Reading classified or help wanted advertisements?	1 2 3 4 5
4. Talking with friends or relatives about possible job leads?	1 2 3 4 5
5. Speaking with previous employers or business acquaintances about possible job leads?	1 2 3 4 5
6. Visiting job fairs?	1 2 3 4 5
7. Contacting employment agencies?	1 2 3 4 5
8. Looking for jobs on the internet?	1 2 3 4 5
9. Making inquiries to prospective employers?	1 2 3 4 5
10. Sending out application letters or filling out job applications?	1 2 3 4 5
11. Going on job interviews?	1 2 3 4 5

## References

[B1] Afua B. B. Lewis P. Mmab M. O. Tessema M. Yoon H. J. (2016). Editorial: training and development in Africa. *Int. J. Train. Dev.* 20 105–106. 10.1111/ijtd.12080

[B2] Ajzen I. (2012). “The theory of planned behavior,” in *Handbook of Theories of Social Psychology* eds Lange P. A. M. Kruglanski A. W. Higgins E. T. (London: Sage) 438–459.

[B3] Arulampalam W. Gregg P. Gregory M. (2001). Unemployment scarring. *Econ. J.* 111 577–584. 10.1111/1468-0297.00663

[B4] Asparouhov T. Muthén B. (2012). *Using Mplus TECH11 and TECH14 to Test the Number of Latent Classes.* Available online at: https://www.Statmodel.com/examples/webnotes/webnote14.pdf (accessed on 4, December 2015)

[B5] Bacaria J. Coll J. M. Sánchez-Montijano E. (2015). *El Mercado Laboral en España: Problemas, Retos y Tendencias de Futuro.* Berlin: Social Inclusion Monitor Europe.

[B6] Bell D. N. F. Blanchflower D. G. (2011). Young people and the great recession. *Oxford Rev. Econ. Pol.* 27 241–267. 10.1093/oxrep/grr011

[B7] Benedicto J. (2013). “De la integración adaptativa al bloqueo en tiempos de crisis. Preocupaciones y demandas de los jóvenes,” in *Actores y Demandas en España. Análisis de un Inicio de Siglo Convulso* ed Morán M. L. (Madrid: Los Libros de la Catarata, Colección Investigación y Debate).

[B8] Berntson E. (2008). *Employability Perceptions: Nature, Determinants, and Implications for Health and Well-being.* Doctoral dissertation, Göteborg: Psykologiska institutionen.

[B9] Blau G. Petrucci T. McClendon J. (2013). Correlates of life satisfaction and unemployment stigma and the impact of length of unemployment on a unique unemployed sample. *Career Dev. Int.* 18 257–280. 10.1108/cdi-10-2012-0095

[B10] Bronk K. C. Leontopoulou S. McConchie J. (2018). Youth purpose during the great recession: a mixed-methods study. *J. Posit. Psychol.* 14 405–416. 10.1080/17439760.2018.1484942

[B11] Caska B. A. (1998). The search for employment: motivation to engage in a coping behavior 1. *J. Appl. Soc. Psychol.* 28 206–224. 10.1111/j.1559-1816.1998.tb01702.x

[B12] Chen D. J. Q. Lim V. K. G. (2012). Strength in adversity: the influence of psychological capital on job search. *J. Organ. Behav.* 33 811–839. 10.1002/job.1814

[B13] Chiesa R. Fazi L. Guglielmi D. Mariani M. (2018). Enhancing substainability: psychological capital, perceived employability, and job insecurity in different work contract conditions. *Sustainability* 10:2475 10.3390/su10072475

[B14] Cifre E. Vera M. Sánchez-Cardona I. de Cuyper N. (2018). Sex, gender identity, and perceived employability among spanish employed and unemployed youngsters. *Front. Psychol.* 9:2467. 10.3389/fpsyg.2018.02467 30581404 PMC6292941

[B15] Crossley C. D. Highhouse S. (2005). Relation of job search and choice process with subsequent satisfaction. *J. Econ. Psychol.* 26 255–268. 10.1016/j.joep.2004.04.001

[B16] De Battisti F. Gilardi S. Guglielmetti C. Siletti E. (2016). Perceived employability and reemployment: do job search strategies and psychological distress matter? *J. Occup. Organ. Psychol.* 89 813–833. 10.1111/joop.12156

[B17] De Cuyper N. De Witte H. (2010). Temporary employment and perceived employability: mediation by impression management. *J. Career Dev.* 37 635–652. 10.1177/089484530935705

[B18] Ellis R. A. Taylor M. S. (1983). Role of self-esteem within the job search process. *J. Appl. Psychol.* 68 632–640. 10.1037/0021-9010.68.4.632

[B19] Fallows S. Steven C. (2000). Building employability skills into the higher education curriculum: a university-wide initiative. *Educ. Train.* 42 75–83. 10.1108/00400910010331620

[B20] Fernández-Valera M. M. (2018). *El Capital Psicológico Como Predictor de la Empleabilidad y la Búsqueda de Empleo en Desempleados Jóvenes*, Tesis doctoral. Murcia: Universidad de Murcia.

[B21] Forrier A. Verbruggen M. De Cuyper N. (2015). Integrating different notions of employability in a dynamic chain: the relationship between job transitions, movement capital and perceived employability. *J. Vocat. Behav.* 89 56–64. 10.1016/j.jvb.2015.04.007

[B22] Georgiou K. Nikolaou I. (2018). The impact of a training intervention developing psychological capital on job search success. *Acad. Manag. Proc.* 2017:15130 10.5465/ambpp.2017.15130abstract

[B23] Gómez D. D. Arévalo M. T. (2013). *La Estrategia de Emprendimiento y Empleo Joven en la Ley 11/2013: Desempleo, Empleo y Ocupación Juvenil.* Albacete: Editorial Bomarzo.

[B24] Harms P. D. Krasikova D. V. Luthans F. (2018). Not me, but reflects me: validating a simple implicit measure of psychological capital. *J. Pers. Assess.* 100 551–562. 10.1080/00223891.2018.1480489 29927679

[B25] Hernández P. M. Méndez M. I. Pedreño A. C. Tovar M. A. (2012). *El Mercado Laboral de los Jóvenes en la Región de Murcia.* Región de Murcia: Consejo Económico y Social.

[B26] Holmstrom A. J. Russell J. C. Clare D. D. (2013). Esteem support messages received during the job search: a test of the CETESM. *Commun. Monogr.* 80 220–242. 10.1080/03637751.2013.775699

[B27] Instituto de la Juventud [INJUVE] (2013). *Situación Actual del Empleo Juvenil en. (España).* Madrid: Ministerio de Sanidad, Servicios Sociales e Igualdad.

[B28] International Labour Organization [ILO] (2013). *Tendencias Mundiales del Empleo Juvenil 2013: Una Generación en Peligro.* Ginebra: International Labour Organization

[B29] International Labour Organization (2015). *Tendencias Mundiales del Empleo Juvenil 2015: Promover la Inversión en Empleos Decentes Para los Jóvenes.* Ginebra: International Labour Organization.

[B30] Kanfer R. Wanberg C. R. Kantrowitz T. M. (2001). Job search and employment: a personality–motivational analysis and meta-analytic review. *J. Appl. Psychol.* 86 837–855. 10.1037/0021-9010.86.5.837 11596801

[B31] Koen J. Klehe U-C. Van Vianen A. E. M. (2013). Employability among the long-term unemployed: a futile quest or worth the effort? *J. Vocat. Behav.* 82 37–48. 10.1016/j.jvb.2012.11.001

[B32] Lim V. K. G. Chen D. Aw S. S. Y. Tan M. (2016). Unemployed and exhausted? Job-search fatigue and reemployment quality. *J. Vocat. Behav.* 92 68–78. 10.1016/j.jvb.2015.11.003

[B33] Lo Presti A. Ingusci E. Magrin M. E. Manuti A. Scrima F. (2019). Employability as a compass for career success: development and initial validation of a new multidimensional measure. *Int. J. Train. Dev.* 23 253–275. 10.1111/ijtd.12161

[B34] Lo Presti A. Pluviano S. (2016). Looking for a route in turbulent waters. *Organ. Psychol. Rev.* 6 192–211. 10.1177/2041386615589398

[B35] López-Kidwell V. Grosser T. J. Dineen B. R. Borgatti S. P. (2013). What matters when: a multistage model and empirical examination of job search effort. *Acad. Manag. J.* 56 1655–1678. 10.5465/amj.2011.0546

[B36] Luthans F. Youssef C. M. Avolio B. J. (2007). *Psychological Capital: Developing the Human Competitive Edge.* Oxford: Oxford University Press.

[B37] Manroop L. Richardson J. (2015). Job search: a multidisciplinary review and research agenda. *Int. J. Manag. Rev.* 18 206–227. 10.1111/ijmr.12066

[B38] McArdle S. Waters L. Briscoe J. P. Hall D. T. (2007). Employability during unemployment: adaptability, career identity and human and social capital. *J. Vocat. Behav.* 71 247–264. 10.1016/j.jvb.2007.06.003

[B39] McKee-Ryan F. Song Z. Wanberg C. R. Kinicki A. J. (2005). Psychological and physical well-being during unemployment: a meta-analytic study. *J. Appl. Psychol.* 90 53–76. 10.1037/0021-9010.90.1.53 15641890

[B40] McQuaid R. W. Lindsay C. (2002). The “Employability Gap”: long-term unemployment and barriers to work in buoyant labour markets. *Environ. Plan. C Govern. Pol.* 20 613–628. 10.1068/c22m

[B41] Meseguer-de Pedro M. Soler-Sánchez M. I. Fernández-Valera M-M. García-Izquierdo M. (2017). Evaluación del Capital Psicológico en trabajadores españoles: diseño y estructura empírica del cuestionario OREA. *Anales de Psicol.* 33 713–721. 10.6018/analesps.33.3.261741

[B42] Miao M. Gan Y. Gan T. Zhou G. (2016). Carry-over effect between diet and physical activity: the bottom-up and top-down hypotheses of hierarchical self-efficacy. *Psychol. Health Med.* 22 266–274. 10.1080/13548506.2016.1160134 26965658

[B43] Morsy H. Jaumotte M. F. (2012). *Determinants of Inflation in the Euro Area: The Role of Labor and Product Market Institutions.* Washington, DC: Fondo Monetario Internacional.ı

[B44] Ngoma M. Dithan Ntale P. (2016). Psychological capital, career identity and graduate employability in Uganda: the mediating role of social capital. *Int. J. Train. Dev.* 20 124–139. 10.1111/ijtd.12073

[B45] Núñez J. L. León J. (2015). Autonomy support in the classroom. *Eur. Psychol.* 20 275–283. 10.1027/1016-9040/a000234

[B46] OECD (2018). *Youth Unemployment Rate (indicator).* Paris: OECD.

[B47] Paul K. I. Moser K. (2009). Unemployment impairs mental health: meta-analyses. *J. Vocat. Behav.* 74 264–282. 10.1016/j.jvb.2009.01.001

[B48] Preacher K. J. Hayes A. F. (2008). Asymptotic and resampling strategies for assessing and comparing indirect effects in multiple mediator models. *Behav. Res. Methods* 40 879–891. 10.3758/brm.40.3.879 18697684

[B49] Ripoll P. Rodríguez I. Hontangas P. Peiró J. M. Prieto F. (1994). “Perspectivas de empleo,” in *Los Jóvenes Ante el Ambiente Laboral y las Estrategias de Adaptación* ed Prieto F. (Valencia: Nau Llibres). 81–87

[B50] Rothwell A. Herbert I. Rothwell F. (2008). Self-perceived employability: construction and initial validation of a scale for university students. *J. Vocat. Behav.* 73 1–12. 10.1016/j.jvb.2007.12.001

[B51] Saks A. M. (2005). “Job search success: a review and integration of the predictors, behaviors, and outcomes,” in *Career Development and Counseling: Putting Theory and Research to Work* eds Brown S. D. Lent R. W. (Hoboken, NJ: John Wiley) 155–79.

[B52] Smith S. A. (2018). “Job searching strategies of millennials,” in *Recruitment, Retention, and Engagement of a Millennial Workforce* ed Smith S.A. (London: Rowman & Littlefield). 1–15.

[B53] Smith S. A. (2019). “Millennial faculty expectations of communication,” in *Leading Millennial Faculty: Navigating the New Professoriate*, ed. Strawser M. G. (London: Lexington Books), 73–86.

[B54] Song Z. Wanberg C. Niu X. Xie Y. (2006). Action–state orientation and the theory of planned behavior: a study of job search in China. *J. Vocat. Behav.* 68 490–503. 10.1016/j.jvb.2005.11.001

[B55] Spector P. E. (2006). Method variance in organizational research. *Organ. Res. Methods* 9 221–232. 10.1177/1094428105284955

[B56] Stumpf S. A. Colarelli S. M. Hartman K. (1983). Development of the career exploration survey (CES). *J. Vocat. Behav.* 22 191–226. 10.1016/0001-8791(83)90028-3

[B57] Sun S. Song Z. Lim V. K. G. (2013). Dynamics of the job search process: developing and testing a mediated moderation model. *J. Appl. Psychol.* 98 771–784. 10.1037/a0033606 23855915

[B58] Vallerand R. J. (1997). Toward a hierarchical model of intrinsic and extrinsic motivation. *Adv. Exp. Soc. Psychol.* 29 271–360 10.1016/s0065-2601(08)60019-2

[B59] Van Hooft E. (2016). *Motivation and Self-Regulation in Job Search: A Theory of Planned Job Search Behavior.* Oxford: Oxford University Press.

[B60] Van Hooft E. Born M. P. Taris T. W. van der Flier H. V. D. Blonk R. W. (2004). Predictors of job search behavior among employed and unemployed people. *Pers. Psychol.* 57 25–59. 10.1111/j.1744-6570.2004.tb02483.x

[B61] Van Hooft E. A. J. Noordzij G. (2009). The effects of goal orientation on job search and reemployment: a field experiment among unemployed job seekers. *J. Appl. Psychol.* 94 1581–1590. 10.1037/a0017592 19916665

[B62] Van Hooft E. A. J. Wanberg C. R. Van Hoye G. (2012). Moving beyond job search quantity. *Organ. Psychol. Rev.* 3 3–40. 10.1177/2041386612456033

[B63] Van Ryn M. Vinokur A. D. (1992). How did it work? An examination of the mechanisms through which an intervention for the unemployed promoted job-search behavior. *Am. J. Commun. Psychol.* 20 577–597. 10.1007/bf00941773 1485612

[B64] Vandekerckhove J. Matzke D. Wagenmakers E.-J. (2015). “Model comparison and the principle of parsimony,” in *Oxford Library of Psychology. The Oxford Handbook of Computational and Mathematical Psychology* eds Busemeyer J. R. Wang Z. Townsend J. T. Eidels A. (New York, NY: Oxford University Press). 300-319

[B65] Vanhercke D. Kirves K. De Cuyper N. Verbruggen M. Forrier A. De Witte H. (2015). Perceived employability and psychological functioning framed by gain and loss cycles. *Career Dev. Int.* 20 179–198. 10.1108/cdi-12-2014-0160

[B66] Verbruggen M. De Vos A. (2019). When people don’t realize their career desires: toward a theory of career inaction. *Acad. Manag. Rev.* 45, 376–394. 10.5465/amr.2017.0196

[B67] Verick S. (2012). Giving up job search during a recession: the impact of the global financial crisis on the south african labour market. *J. Afr. Econ.* 21 373–408. 10.1093/jae/ejr047 28159496

[B68] Vinokur A. Caplan R. D. (1987). Attitudes and social support: determinants of job-seeking behavior and well-being among the unemployed. *J. Appl. Soc. Psychol.* 17 1007–1024. 10.1111/j.1559-1816.1987.tb02345.x

[B69] Vuolo M. Mortimer J. T. Staff J. (2013). Adolescent precursors of pathways from school to work. *J. Res. Adoles.* 24 145–162. 10.1111/jora.12038 24791132 PMC4004182

[B70] Wanberg C. R. Glomb T. M. Song Z. Sorenson S. (2005). Job-search persistence during unemployment: a 10-wave longitudinal study. *J. Appl. Psychol.* 90 411–430. 10.1037/0021-9010.90.3.411 15910139

[B71] Wanberg C. R. Kanfer R. Rotundo M. (1999). Unemployed individuals: motives, job-search competencies, and job-search constraints as predictors of job seeking and reemployment. *J. Appl. Psychol.* 84 897–910. 10.1037/0021-9010.84.6.897 10639909

[B72] Wanberg C. R. Zhu J. van Hooft E. A. J. (2010). The job search grind: perceived progress, self-reactions, and self-regulation of search effort. *Acad. Manag. J.* 53 788–807. 10.5465/amj.2010.52814599

[B73] Yizhong X. Lin Z. Baranchenko Y. Lau C. K. Yukhanaev A. Lu H. (2017). Employability and job search behavior: a six-wave longitudinal study of Chinese university graduates. *Employ. Relat.* 39 223–239. 10.1108/er-02-2016-0042

